# Ginsenoside Rb1 Lessens Gastric Precancerous Lesions by Interfering With β-Catenin/TCF4 Interaction

**DOI:** 10.3389/fphar.2021.682713

**Published:** 2021-09-14

**Authors:** Jinhao Zeng, Xiao Ma, Ziyi Zhao, Yu Chen, Jundong Wang, Yanwei Hao, Junrong Yu, Zhongzhen Zeng, Nianzhi Chen, Maoyuan Zhao, Jianyuan Tang, Daoyin Gong

**Affiliations:** ^1^ Department of Chinese Internal Medicine, Hospital of Chengdu University of Traditional Chinese Medicine, Chengdu, China; ^2^ TCM Regulating Metabolic Diseases Key Laboratory of Sichuan Province, Hospital of Chengdu University of Traditional Chinese Medicine, Chengdu, China; ^3^ School of Pharmacy, Chengdu University of Traditional Chinese Medicine, Chengdu, China; ^4^ Department of Pathology, Hospital of Chengdu University of Traditional Chinese Medicine, Chengdu, China

**Keywords:** ginsenoside Rb1, gastric precancerous lesions, intestinal metaplasia, dysplasia, β-catenin/TCF4 interaction

## Abstract

**Background:** Seeking novel and effective therapies for gastric precancerous lesions (GPL) is crucial to reducing the incidence of gastric cancer. Ginsenoside Rb1 (GRb1) is a major ginsenoside in ginseng and has been proved to possess multiple bioactivities. However, whether GRb1 could protect against GPL and the underlying mechanisms have not been explored.

**Methods:** We evaluated the effects of GRb1 on gastric precancerous lesions in rats on macroscopic, microscopic and ultramicroscopic levels. Then, an antibody array was employed to screen differential expression proteins (DEPs). Validation for the targeting DEP and investigation for the possible mechanism was conducted using immunohistochemistry, qRT-PCR, TUNEL apoptosis assay, immunoprecipitation and immunoblotting.

**Results:** GRb1 was found to reverse intestinal metaplasia and a portion of dysplasia in the MNNG-induced GPL rats. The antibody array assay revealed seven DEPs in GPL rats as compared to control rats (5 DEPs were up-regulated, while two DEPs were down-regulated). Among the DEPs, β-catenin, beta-NGF and FSTL1 were significantly down-regulated after GRb1 administration. Our validation results revealed that enhanced protein expression and nuclear translocation of β-catenin were present in animal GPL samples. In addition, analysis of human gastric specimens demonstrated that β-catenin up-regulation and nuclear translocation were significantly associated with advanced GPL pathology. GRb1 intervention not only decreased protein expression and nuclear translocation of β-catenin, but interfered with β-catenin/TCF4 interaction. Along with this, declined transcriptional and protein expression levels of downstream target genes including c-myc, cyclin D1 and Birc5 were observed in GRb1-treated GPL rats.

**Conclusion:** GRb1 is capable of preventing the occurrence and progression of GPL, which might be contributed by diminishing protein expression and nuclear translocation of β-catenin and interfering with β-catenin/TCF4 interaction.

## Introduction

Gastric cancer still remains a major cancer worldwide, ranking fifth for incidence and fourth for mortality globally in the latest published global cancer statistics (Sung et al., 2021). Gastric carcinogenesis is a multistep process, in which gastric intestinal metaplasia (IM) and dysplasia (DYS) precedes most gastric adenocarcinomas and hence they are widely recognized as precancerous lesions (GPL). It is well established that the risk of gastric cancer increases with the severity of precancerous lesions (Akbari et al., 2019). This underscores the importance of screening, surveillance and treatment of patients harboring precancerous lesions. Preventing and even reversing the progression of GPL would definitely provide tangible benefits in reduction of gastric cancer incidence (De Vries et al., 2008; Akbari et al., 2019). For the therapy of GPL, it is recognized at present that endoscopic mucosal dissection was recommended for severe dysplasia and early gastric cancer (Pimentel-Nunes et al., 2019); however, no specific treatment for a majority of GPL available in clinical practice. Some vitamin and mineral supplements, particularly folic acid (Zhu et al., 2003), may be beneficial in reducing risk of progression to gastric cancer, but their therapeutic effect against GPL has not been verified (Jacobs et al., 2002; Dawsey et al., 2014). Seeking novel and effective therapies for patients with GPL has fueled a huge concern.

Ginsenoside Rb1 (known as one of the main bioactive components of *ginseng*) is the major constituent screened from Weipixiao (Zeng et al., 2016), a Chinese herbal prescription showing therapeutic effect against GPL, as revealed by previous clinical and animal studies (He et al., 2017; Zhang, 2017; Zeng et al., 2018). Previously, GRb1 has been proved to be a safe extraction and possess antineoplastic (Liu et al., 2017), antioxidative (Miao et al., 2017), anti-inflammatory (Liu et al., 2020) and anti-angiogenesis (Lu et al., 2017) bioactivities. In addition, an earlier study identified the gastroprotective activity of GRb1 in an ethanol-induced gastric lesion model, and demonstrated that the anti-ulcer effect is produced through an increase in mucus secretion (Jeong et al., 2003). GRb1 was recently found to reduce intestinal histological injury, and suppress inflammatory responses and oxidative stress (Chen et al., 2019). Moreover, GRb1 promote intestinal epithelial wound healing in a colitis rat model through regulating extracellular signal-regulated kinase and Rho signaling (Toyokawa et al., 2019). However, heretofore, whether GRb1 is capable of halting and even reversing GPL still remains unknown.

The Wnt/β-catenin signaling pathway plays a key role in the regulation of cell proliferation, differentiation, embryogenesis and tumorigenesis. The contribution of aberrant Wnt/β-catenin pathway to development of gastric cancer has been established (Clevers, 2006; Hanaki et al., 2012). Wnt/β-catenin pathway has become the new targets for anti-tumor therapy, for instance, disruption of the pathway could suppress metastatic activity in gastric cancer cells (Hanaki et al., 2012). β-catenin serves as the key downstream effector of the canonical pathway. When Wnt ligands binds to its membrane receptor complex, β-catenin becomes stabilized and accumulates in cytoplasm, and then enters to nucleus with the TCF, thereby driving the transcription of multiple proliferation- and apoptosis-associated genes (Nusse and Clevers, 2107). In this process, β-catenin/TCF4 interaction is a central variable. The downstream target genes of Wnt/β-catenin, such as c-Jun, c-Myc and cyclin D1, Birc5 and Wisp1, have a significant relevance to multiple cellular biological behaviors including proliferation and apoptosis. Studies have shown that when the expression of β-catenin is reduced, the expressions of its activation targets including c-Jun, c-Myc and cyclin D1 are also decreased (Qin et al., 2019). Up-regulation of Birc5, which would promote cell proliferation, is activated by Wnt/β-catenin signaling pathway (Fujiya et al., 2020). Additionally, activated Wnt/β-catenin signaling promotes cancer metastasis through paracrine Wisp1 (Tai et al., 2014).

We verified the hypothesis in the present study that GRb1 could protect against gastric precancerous lesions. Next, we employed an antibody array to screen differential expression proteins and then carried out validation experiments. Finally, we sought to investigate the possible underlying mechanism regarding regulation on nuclear translocation of β-catenin and β-catenin/TCF4 interaction. Our work may provide experimental evidence for potential clinical use of GRb1 in GPL treatment.

## Materials and Methods

### Animals

Sprague-Dawley rats weighing 160–180 g (half male and half female) were obtained from Chengdu Dashuo Experimental Animal Co., Ltd., Chengdu, China (SCXK-2020-030). This experiment was conducted in TCM Regulating Metabolic Diseases Key Laboratory of Sichuan Province, Hospital of Chengdu University of Traditional Chinese Medicine. Animal welfare considerations taken and all procedures were approved by the Institutional Animal Care and Use Committee (approval no. 2019-17).

### Clinical Tissue Samples

Formalin-fixed, paraffin-embedded samples of GPL gastric mucosa (94 cases) and normal gastric mucosa (85 cases) were provided from the Hospital of Chengdu University of TCM (Chengdu, China), and retrospectively analyzed. The pathological diagnosis of each sample was performed by two independent senior pathological staffs in the Department of Pathology. This study was approved by the Institutional Review Board of the Teaching Hospital of Chengdu University of TCM (Chengdu, China) (approval no. 2018KL-023) and written informed consent was obtained from all participants for being included in the study.

### Drugs and Reagents

N-methyl-N′-nitro-N-nitrosoguanidine (MNNG) was purchased from Tokyo Chemical Industry CO., LTD., Japan (cat. no. M0527); Ginsenoside Rb1 was supplied by Hefei Bomei Biotechnology CO., LTD., China (cat. no. BZP0234); Rat antibody array 90 glass slide kit was supplied by RayBiotech, United States (cat. no. AAR-BLG-1); β-catenin antibody was purchased from Thermo Fisher Scientific, Inc., United States (cat. no. MA1-301); Transcription factor 7-like 2 (TCF-4/TCF7L2) antibody, c-myc antibody, cyclin D1 antibody, baculoviral IAP repeat containing 5 (Birc5) antibody and proliferating cell nuclear antigen (PCNA) antibody were purchased from abcam, United Kingdom (cat. nos. ab134275; ab32072; ab134175; ab76424; ab18197); Wnt1-inducible signaling pathway protein 1 (Wisp1) antibody, and c-jun antibody were obtained from Bioss, Inc., China (cat. nos. bs-6321R; bs-0670R). Ki67 antibody was supplied by Novus Biologicals, LLC, United States (cat. no. NB500-170). TdT-mediated dUTP Nick-End Labeling (TUNEL) apoptosis assay kit was supplied by Boster Biological Technology CO., LTD., China (cat. no. MK1025).

### GPL Model and GRb1 Treatment

**FIGURE 1:**
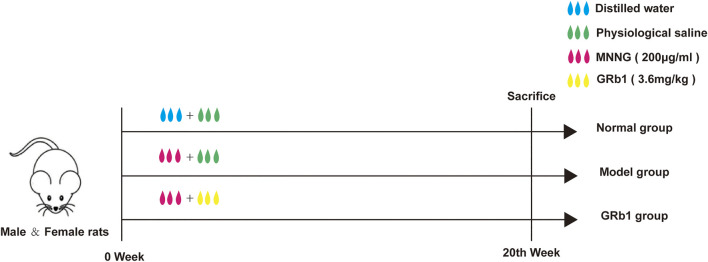
Experimental flow chart of MNNG-induced animal model and GRb1 treatment. **(A)** Normal group (treated with distilled water and physiological saline); **(B)** Model group (treated with MNNG and physiological saline; model group); **(C)** GRb1 group (treated with MNNG and GRb1). **Abbreviations:** MMNG, N-methyl-N′-nitro-N-nitrosoguanidin; GRb1, ginsenoside Rb1.

Animals were randomly assigned to three groups as follows (*n* = 10): 1) Normal (treated with distilled water and physiological saline, normal group); 2) Model (treated with MNNG and physiological saline; model group); 3) Model + GRb1 (treated with MNNG and GRb1; GRb1 group). MNNG is a carcinogen, which can induce the occurrence of gastric precancerous lesions (Saito et al., 1970; Tatematsu et al., 1988). To establish GPL model, the animals, except the normal controls, were allowed to drink MNNG solution (200 μg/ml) *ad libitum*, and underwent hunger-satiety shift every other day. The modeling procedure lasted for 20 consecutive weeks. Simultaneously, from the first day of MNNG treatment to the 20th week, GRb1 at 3.6 mg/kg was intragastrically administered to the rats in the GRb1 group once a day, while rats in normal group and model group received vehicle physiological saline by gastrogavage (10 ml/kg) once daily. At the end of 20th week, all animals were humanely anesthetized with sodium pentobarbital (140 mg/kg i. p.) after 12 h fasting. Following sacrifice by cervical dislocation, stomachs were harvested immediately. The flow chart of the experiment is shown in Figure 1.

### Hematoxylin and Eosin Staining

Gastric tissues were fixed in 4% paraformaldehyde overnight at room temperature, dehydrated in graded ethanol series (30, 50, 70, 95 and 100%) and then embedded in paraffin. To examine pathological morphology, paraffin-embedded samples were cut into 5 μm-thick sections and then stained with hematoxylin and eosin. Each slide was monitored under microscopy (IX71; Olympus Corporation) and images were captured. The magnifications used were ×400. These slides were evaluated by two independent pathologists in a blinded manner.

### Transmission Electron Microscope

Fresh gastric mucosa was cut into 1 mm^3^ blocks and fixed in 2.5% glutaraldehyde in cacodylate buffer (pH 7.4) at 4°C for 4 h, and then post-fixed in 1% osmium tetroxide, also in phosphate buffer for 2 h. Next, samples were routinely dehydrated in graded ethanol series (50, 70, 80, 90, 95 and 100%) and transferred to acetone solution for 20 min. For infiltration, the samples were immersed in a mixture of acetone and epoxy resin twice (2:1 for 3 h at the first time, 1:2 for overnight at the second time), and then embedded in epoxy resin-filled capsules. Finally, 70 nm ultrathin sections were counterstained with 2% aqueous uranyl acetate and 0.8% lead citrate. Ultrastructure of gastric epithelial cells was observed and photographed using a transmission electron microscope (H-7650; Hitachi Ltd.). The magnification was ×10,000.

### Alcian Blue-Periodic Acid Schiff and High Iron Diamine Staining

AB-PAS staining was performed to evaluate the severity of IM lesion. Neutral mucins present in normal mucosa were stained magenta, while acidic mucins present in IM lesion were stained blue. Then, HID-PAS staining was conducted to further assess small intestinal-type metaplasia (S-IM) and colonic-type metaplasia (C-IM), in which sulfomucins expressed only in C-IM lesion were stained brown. Images were captured under microscopy (IX71; Olympus Corporation) with magnification of ×100.

### Antibody Array Assay

Biotin label-based rat antibody array was conducted to determine the expression levels of 90 rat proteins, according to the manufacturer’s instructions. Briefly, gastric epithelium was collected and homogenized in pre-cooled lysis buffer containing protease inhibitor cocktail at 4°C. Homogenates were then centrifuged at 12,000 rpm for 15 min twice. Next, supernatants were dialyzed in separate dialysis tubes, followed by determination of the total protein concentration using the bicinchoninic acid (BCA) assay (cat. no. 23227, Pierce Scientific Rockford, United States). After biotin-labeling the samples and blocking process, 400 μl of diluted samples was added into each well for incubation overnight at 4°C. Then, each sub-array was incubated with Cy3-Conjugated Streptavidin at room temperature for 2 h (avoid exposure to light). Finally, the glass slides were scanned to detect the fluorescent signals of microarrays using an InnoScan 300 Microarray Scanner (Innopsys, France) at a wavelength of 532 nm.

### Immunohistochemistry

Paraffin-embedded gastric tissues were cut into 3 µm-thick sections, and dewaxed with xylene at room temperature and rehydrated in a descending ethanol series (100, 95, 85 and 75%). For antigen retrieval, sections were heated at 97°C for 20 min. Following a peroxidase blocking with 3% hydrogen peroxide for 15 min and 5% bovine serum albumin blocking for 30 min, the sections were incubated with primary antibodies against β-catenin (1:500), TCF-4 (1:1,000), PCNA (1:500) and Ki67 (1:100) overnight at 4°C. The sections were then exposed to HRP/Fab polymer conjugate at room temperature for 30 min (cat. no. PV-6000-D, Zhongshan Goldenbridge Biotechnology Co., Ltd.), following which they were stained with 3,3′-diaminobenzidine solution for 5 min and counterstained with hematoxylin for 20 s at room temperature. Three random visual fields were selected and photographed from each section. Quantification of expression levels was determined by mean of integrated optical density (IOD) using Image Pro Plus 6.0 software (Media Cybernetics, Inc.). Moreover, the expression pattern of β-catenin and its relationships with clinicopathological characteristics of GPL patients were evaluated. The sections were assessed by two independent investigators, without prior knowledge of the clinicopathological data, in a blinded manner. The immunoreactivity scores (IRS) were determined by the sum total of the percentage of positive cells (0 points, 0–5% positive cells; 1 point, 6–25%; 2 points, 26–50%; 3 points, 51–75% and 4 points, 76–100%), and staining intensity scores (0 points, no staining; 1 point, weak staining; 2 points, moderate staining and 3 points, strong staining). A final IRS ≥4 indicated strong positivity, while scores <4 indicated weak positivity.

### TUNEL Apoptosis Assay

TUNEL apoptosis assay was carried out according to the manufacturer’s instructions. Briefly, 3 µm-thick sections, sliced from paraffin-embedded gastric tissues, were routinely dewaxed and rehydrated. The sections were incubated with proteinase K working solution at 37°C for 15 min, followed by incubation with labeling buffer containing Terminal deoxynucleotidyl Transferase (TdT) and digoxin labeled deoxyuridine triphosphate (dUTP). Next, proceed with blocking reagent prior to incubation with biotinylated anti-digoxin antibody at 37°C for 30 min. Finally, samples were exposed to strept avidin-biotin complex (SABC), followed by staining with 3, 3′-diaminobenzidine solution and counterstaining with hematoxylin. Apoptotic cells were shown as nuclear staining. Counting for apoptotic cells was performed using light microscopy (IX71; Olympus Corporation) with magnification of ×200. Three randomly selected fields were captured for each section, in which apoptosis index was calculated.

### Immunoprecipitation and Immunoblotting

Frozen gastric epithelium was collected, and homogenates and supernatants were prepared as described above. Total protein concentration was determined using the BCA assay (cat. no. 23227, Pierce Scientific Rockford, United States). Then, 480 μg of total protein were incubated with 20 μl of protein A/G beads (cat. no. sc-2003; Santa Cruz Biotechnology, Inc.) at 4°C for 1 h (the negative controls were incubated with 20 μl of protein A/G beads and 2 μl lgG). After centrifugation, 2 μl β-catenin antibody (1:200) was added in lysate supernatants for incubation overnight at 4°C, and, next, 20 μl of protein A/G beads was mixed, followed by gentle rotation at 4°C for 2 h. Following centrifugation and removal of supernatants, the beads were collected. The immunoprecipitation complex was washed 4 times, mixed with reduced loading buffer, boiled, and centrifuged. Finally, western blotting was performed to detect the abundance of both β-catenin and TCF-4 proteins in the immunoprecipitation complex. To determine the protein expression levels of c-myc, cyclin D1, Wisp1 and Birc5, western blotting was also performed as described previously (Li et al., 2021).

### Reverse Transcription and Quantitative Real-Time PCR

Total RNA from gastric epithelium was extracted using a TRIzol kit (G3013; Servicebio). RNA concentration and quality were determined using the spectrophotometer (NanoDrop 2000; Thermo Fisher Scientific Inc.) and 1% agarose gels. Complementary DNA was synthesized using the RevertAid First Strand cDNA Synthesis Kit (K1621; Thermo Fisher Scientific Inc.). Subsequently, mRNA levels of *c-myc, c-jun, cyclin D1*, *Wisp1* and *Birc5* were determined using StepOne Plus real-time PCR System (Applied Biosystems Inc.). Thermal conditions were as follows: 10 min at 95°C, 40 cycles of 15 s at 95°C and 60 s at 60°C, with 0.3°C rise per 15 s from 60°C to 95°C. *Gapdh* was applied as an endogenous control for RNA input. Differences in amplification were calculated using the 2^−△△Ct^ method. The primer sequences used were as follows: *c-myc* forward 5′-AAA​ACC​CGA​CAG​TCA​CGA​CG-3′ and reverse 5′-GTA​GCG​ACC​GCA​ACA​TAG​GAC-3′; *cyclin D1* forward 5′-TTC​ATC​GAA​CAC​TTC​CTC​TCC​A-3′ and reverse 5′-GAGGGTGGGTTGGAAA TGAA-3′; *c-jun* forward 5′-GCA​ATG​GGC​ACA​TCA​CCA​CTA​C-3′ and reverse 5′-GTG​ACA​CTG​GGC​AGC​GTA​TTC​T-3′; *Wisp1* forward 5′-ACATCAAGGCAGG GAAGAAATG-3′ and reverse 5′-CCT​CTG​GAC​ACT​GGA​AAT​CAA​C-3′; *Birc5* forward 5′-GAC​CAC​CGG​ATC​TAC​ACC​TTC-3′ and reverse 5′-CTCGGTAGGGCA GTGGATGAA-3′; *Gapdh* forward 5′-CTG​GAG​AAA​CCT​GCC​AAG​TAT​G-3′ and reverse 5′-GGT​GGA​AGA​ATG​GGA​GTT​GCT-3′.

### Statistical Analysis

For antibody array assay, R software (version 3.6.3) package “limma” was employed to analyze the differential expression proteins using a linear model of empirical Bayesian method. When *p* value < 0.05, differential expression is considered. The other statistical data was analyzed using SPSS 23.0 software (IBM Inc.). Differences between groups were evaluated using one-way analysis of variance, followed by Tukey method for homogeneous data and Dunnett’s T3 method for non-homogeneous data. Data are expressed as the mean ± standard deviation. *p* < 0.05 was considered to indicate a statistically significant difference. Unpaired Student’s t-test was used to assess the differences in β-catenin expression levels between gastric precancerous lesions group and control group. Pearson’s χ^2^ test and Fisher’s exact test were used to assess the association between nuclear localization of β-catenin, β-catenin expression and clinicopathological characteristics.

## Results

### GRb1 Ameliorates Pathomorphology of Dysplasia

**FIGURE 2:**
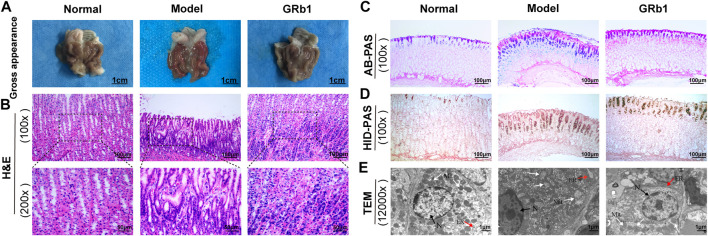
Effects of GRb1 on pathomorphology of dysplasia and on gastric intestinal metaplasia in GPL model rats. **(A)** Gross evaluation of the gastric mucosa. **(B)** Microscopic appearance of the gastric epithelium using hematoxylin and eosin staining (magnification ×100, ×200). **(C)** Evaluation for intestinal metaplasia lesion using alcian blue-periodic acid schiff staining (magnification ×100). **(D)** Assessment for colonic-type metaplasia using high iron diamine-periodic acid schiff staining (magnification ×100). **(E)** Representative images indicating the ultrastructures of epithelial cells using transmission electron microscope (TEM) (magnification ×12,000). **Abbreviations:** GRb1, ginsenoside Rb1; GPL, gastric precancerous lesions. N, nucleus (black arrow); Mit, mitochondrion (white arrow); ER, endoplasmic reticulum (red arrow).

We test the effects of GRb1 on pathomorphology of gastric epithelium in GPL rats on macroscopic, microscopic and ultramicroscopic levels. As shown in Figures 2A,B,E, normal control rats exhibited normal macroscopic appearance of gastric mucosa, intact arrangement and morphology of gland and cells under light microscope, as well as intact and clear ultrastructure of epithelial cells revealed by TEM. By contrast, gastric mucosa from GPL model rats appeared as dark red, poor lustrousness, and, often, rough-surfaced. Light microscope revealed distorted, crowded glands in which cellular atypia characterized by enlarged and hyperchromatic nuclei, increased nuclear-cytoplasmic ratio and loss of polarity was noted. In addition, TEM provided evidence of pleomorphic nuclei, prominent chromatin condensation and nuclear membrane invagination in the GPL model rats. Swollen mitochondria with broken cristae, and expanded endoplasmic reticulum with sharp decreased numbers of ribosomes were also present. These observations were suggestive of DYS lesion. In most cases of GRb1-treated rats, the appearances of these aberrant morphologic alterations were less pronounced than those in GPL model rats. Therefore, GRb1 efficiently ameliorated the pathological morphologies of DYS lesion in GPL rats.

### GRb1 Effectively Halts and Even Reverses Gastric Intestinal Metaplasia

We then examined the degree of IM lesion in gastric epithelium using AB-PAS staining. Neutral mucins present in normal gastric epithelium were stained magenta, while acidic mucins present in IM lesion were stained blue (or purple when combined with neutral). As depicted in Figure 2C, blue- or purple-stained foci were found in gastric epithelium from model rats, suggesting that extensive IM lesion appeared. Acidic mucins can be, in turn, sialic or sulfated; the latter stain brown with HID (19). We thus combined HID-PAS staining to further assess small intestinal-type metaplasia (S-IM) and colonic-type metaplasia (C-IM). In model rats, brown-stained sulfomucins appeared and, in approximately two-thirds, became the predominant mucin, indicating the presence of C-IM lesion, as shown in Figure 2D. Comparatively, both types of sulfomucins and sialomucins were becoming less abundant in GRb1-treated rats. These findings indicated that GRb1 effectively halted and even reversed both S-IM and C-IM lesions.

### Differential Expression Proteins in GPL Model Group and Effect of GRb1 on DEPs

**FIGURE 3:**
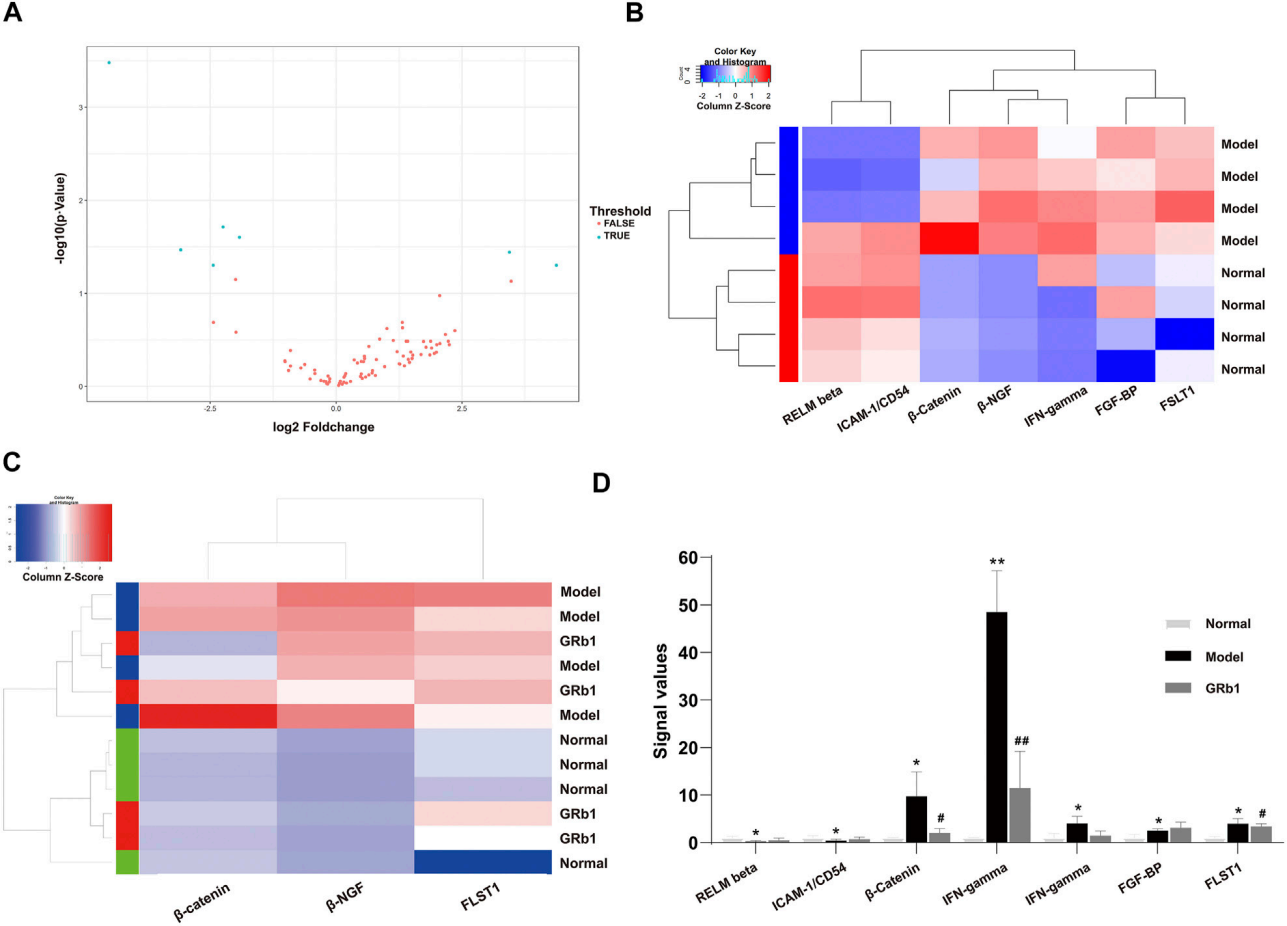
Identification of differential expression proteins (DEPs) in GPL model rats using antibody array assay and the effects of GRb1 on DEPs. **(A)** Volcano plot indicating the distribution of all proteins between the normal and model groups. **(B)** Heatmap representation showing the seven DEPs between the normal and model groups, in which 5 DEPs were significantly up-regulated, while two DEPs were significantly down-regulated. **(C)** Heatmap representation indicating the three DEPs among the three groups (normal, model and GRb1 groups). **(D)** Histograms showing signal values of the three DEPs among the three groups (normal, model and GRb1 groups). *p < 0.05 and **p < 0.01 vs. Normal group. ^#^ *p* < 0.05 and ^##^ *p* < 0.01 vs. Model group. Data are presented as mean ± SEM (*n* = 4). **Abbreviations:** DEPs, differential expression proteins; GRb1, ginsenoside Rb1; GPL, gastric precancerous lesions; SEM, standard error of mean.

To screen differential expression proteins (DEPs) in the GPL rats relative to normal controls, we carried out antibody array assay. Statistical analysis results unveiled that, compared to the normal group, seven DEPs were screened out in the GPL model group (Figure 3A). Among the seven DEPs, five proteins (beta-nerve growth factor (NGF), interferon (IFN)-gamma, fibroblast growth factor-binding protein (FGF-BP), follistatin-like 1 (FSTL1) and β-catenin) were significantly up-regulated; while two proteins (resistin-like molecule (RELM) beta and intercellular adhesion molecule-1 (ICAM-1)/CD54) were remarkably down-regulated (Figure 3B). It is noteworthy that, among the five proteins up-regulated in the GPL model group, β-catenin, beta-NGF and FSTL1 were significantly down-regulated after GRb1 administration (Figures 3C,D). Intriguingly, the result was in tune with our earlier study showing that an herbal formula Weipixiao, of which GRb1 is the major bioactive constituent (Zeng et al., 2016) could down-regulate β-catenin expression in GPL rats. Thus, this strongly implies that β-catenin may be a potential therapeutic target in GRb1-induced therapeutic effects on GPL.

### Verification of β-Catenin Up-Regulation and Nuclear Translocation in Human GPL Specimens

**FIGURE 4:**
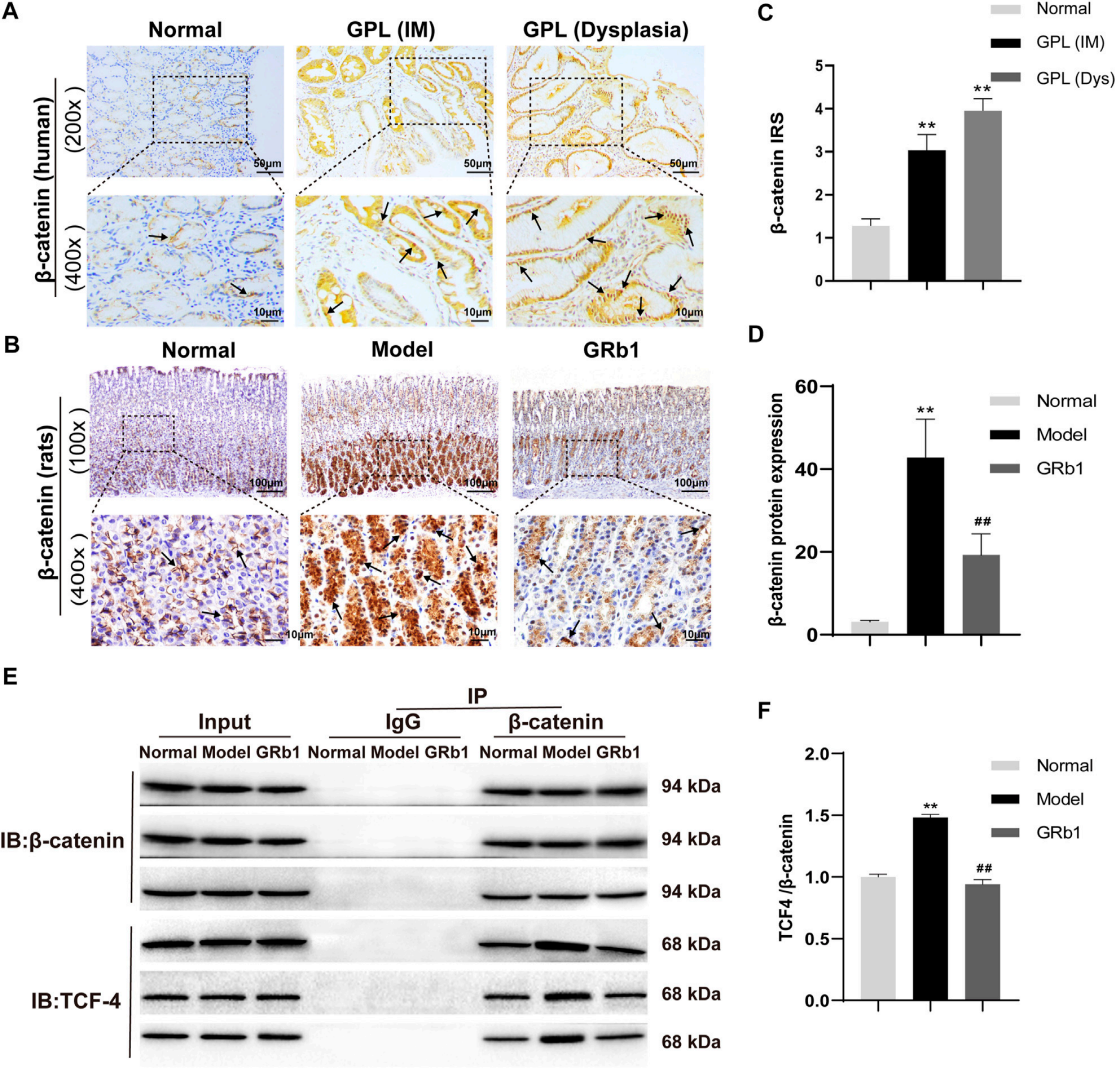
Expression patterns of β-catenin in both human and animal GPL specimens, and the effects of GRb1 on β-Catenin/TCF4 interaction in GPL model rats. Representative IHC images demonstrating the expression localization of β-catenin in epithelial epithelium from **(A)** human specimens (magnification ×200, ×400) and **(B)** animal samples (magnification ×100, ×400). **(C)** Semi-quantitative analysis of β-catenin protein expression in human specimens (*n* = 179). **(D)** Semi-quantitative analysis of β-catenin protein expression levels in animal samples (*n* = 10). **(E)** Representative bands illustrate the β-Catenin/TCF4 interaction. Immune precipitation for β-catenin was performed with an IgG antibody as a control, and then TCF4 was detected using western blotting. **(F)** Quantification of the intensities of western blotting bands (*n* = 6). *p < 0.05 and **p < 0.01 vs. Normal group. ^#^ *p* < 0.05 and ^##^ *p* < 0.01 vs. Model group. Data are presented as mean ± SEM. **Abbreviations:** GRb1, ginsenoside Rb1; GPL, gastric precancerous lesions; TCF4, transcription factor 7-like two; IHC, immunohistochemistry; SEM, standard error of mean.

In order to verify the role of β-catenin in precursors of gastric cancer, we investigate the expression and subcellular localization of β-catenin in 94 cases of human GPL specimens and in 85 cases of gastric specimens from the healthy. β-catenin expression was identified in 80.9% (76/94) of the GPL specimens and 72.9% (62/85) of the normal specimens. The β-catenin immunoreactivity was notably stronger in the human GPL specimens than in the healthy controls (Figures 4A,C). In the normal specimens from the healthy, moreover, the proportions of β-catenin localization at cell membrane/cytoplasm and nucleus were 95.2% (59/62) and 4.8% (3/62), respectively. By contrast, incidence of β-catenin localization at cell membrane/cytoplasm decreased (30/76, 39.5%) but significantly increased at the nucleus (46/76, 60.5%) in the human GPL specimens.

Association between clinical characteristics and β-catenin positivity/subcellular localization in patients with GPL was investigated. Our analysis demonstrated that rate of nuclear localization of β-catenin was significantly different between normal gastric epithelium and GPL samples. Moreover, nuclear expression of β-catenin in gastric epithelium increased depending on the pathological grade of GPL (From small intestinal-type metaplasia to severe dysplasia). In the GPL specimens assessed, high percentage of nuclear staining with β-catenin was not significantly associated with age, sex and location of lesion (Table 1). Analysis of the association between β-catenin expression levels (strong positivity vs. weak positivity/absent) and clinicopathological characteristics demonstrated that strong β-catenin positivity was significantly associated with advanced GPL pathology. In the 94 cases of gastric precancerous lesions assessed, high β-catenin expression was not associated with gender, location of lesion and Hp infection (Table 2).

**TABLE 1:** Correlation between nuclear localization of β-catenin and clinical characteristics.

Variable	Case	Membrane/cytoplasm	Nucleus	*p*-value, nucleus vs. membrane/cytoplasm
**Age**				
<60	49	19	30	0.867
≥60	27	11	16	
**Gender**				
Male	42	17	25	0.842
Female	34	13	21	
**Location of lesion**				
Body	10	3	7	0.850
Angle	16	7	9	
Antrum	38	16	22	
Multiple	12	4	8	
**Hp infection**				
Negative	31	11	20	0.555
Positive	45	19	26	
**Histopathological category**				
Normal gastric epithelium	62	59	3	<0.001
Gastric precancerous lesions	76	30	46	
Small intestinal-type metaplasia	22	15	7	
Colonic-type metaplasia	20	8	12	
Mild dysplasia	19	5	14	0.010
Moderate dysplasia	7	1	6	
Severe dysplasia	8	1	7	

**TABLE 2:** Correlation between β-catenin positivity and clinicopathological characteristics of patients with gastric precancerous lesions.

Variable	Case	β-catenin strong positivity	β-catenin weak positivity/absent	*p*-value, strong vs. weak/absent
**Age**				
<60	61	39	22	0.545
≥60	33	19	14	
**Gender**				
Male	49	32	17	0.453
Female	45	26	19	
**Location of lesion**				
Body	13	6	7	0.068
Angle	19	16	3	
Antrum	48	26	22	
Multiple	14	10	4	
**Hp infection**				
Negative	40	25	15	0.891
Positive	54	33	21	
**Histopathological category**				
Normal gastric epithelium	85	39	46	0.034
Gastric precancerous lesions	94	58	36	
Small intestinal-type metaplasia	30	17	13	
Colonic-type metaplasia	24	11	13	
Mild dysplasia	23	19	4	0.096
Moderate dysplasia	8	6	2	
Severe dysplasia	9	5	4	

### GRb1 not Only Decreases Protein Expression and Nuclear Translocation of β-Catenin, but Interferes With β-Catenin/TCF4 Interaction

Our verification results in the present animal study showed that β-catenin protein expression was increased and β-catenin preferentially accumulated in the cytoplasm and, in some cases, translocated into the nucleus in GPL model rats (Figure 4B). In accordance with the results of the antibody array, GRb1 decreased protein expression and nuclear translocation of β-catenin (Figure 4D). β-catenin has been widely acknowledged as a key mediator in Wnt pathway in human gastric cancer (Flanagan et al., 2017). Following by cytosol accumulation and nuclear translocation, β-catenin binds to transcriptional factors the T-cell factor (TCF) to induce the transcription of Wnt target genes (Nusse and Clevers, 2107). To check whether GRb1 affected the β-catenin/TCF4 interaction, gastric epithelium was subjected to immunoprecipitation for β-catenin with IgG antibody as a control, and the resulting immunocomplexes were immunoblotted for TCF4. The results revealed that GRb1 effectively diminished the binding of β-catenin to TCF4 (Figures 4E,F), suggesting that GRb1 was able to disrupt the interaction of β-catenin with TCF4.

### GRb1 Lowers Transcriptional and Protein Expression Levels of Downstream Target Genes, Including c-Myc, Cyclin D1 and Birc5

**FIGURE 5:**
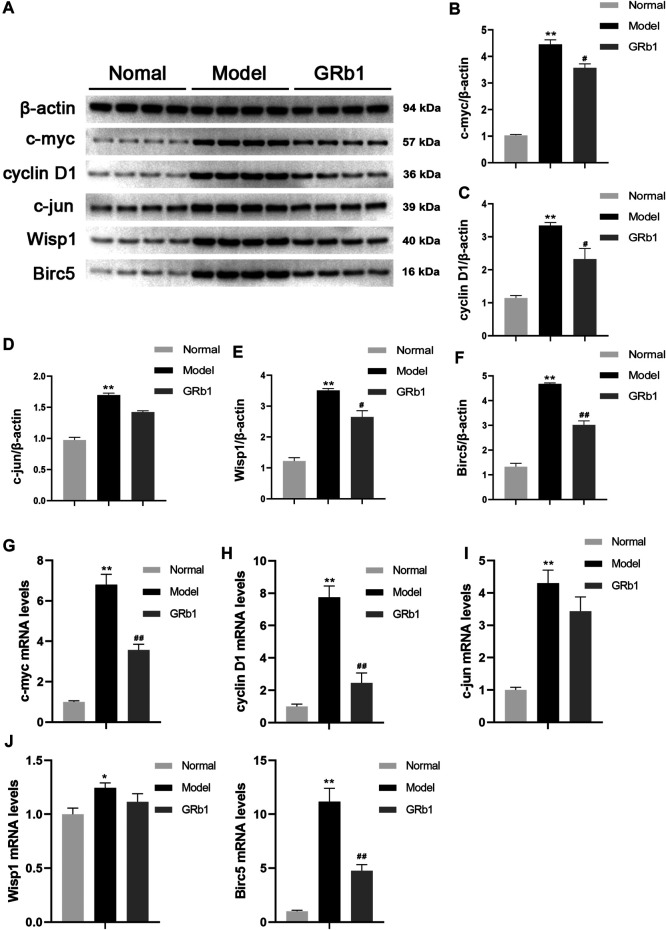
**(A)** Effects of GRb1 on the protein expression levels of c-myc, cyclin D1, c-jun, Wisp1 and Birc5 in GPL model rats. **(A)** Representative western blotting bands of c-myc, cyclin D1, c-jun, Wisp1 and Birc5. Quantitative analysis of **(B)** c-myc, **(C)** cyclin D1, **(D)** c-jun, **(E)** Wisp1, and **(F)** Birc5 in western blotting bands (*n* = 4). Effects of GRb1 on mRNA levels of c-myc, cyclin D1, c-jun, Wisp1 and Birc5 in GPL model rats. Quantization for mRNA levels of **(G)** c-myc, **(H)** cyclin D1, **(I)** c-jun, **(J)** Wisp1, and **(K)** Birc5 in gastric epithelium from each group (*n* = 6).*p < 0.05 and **p < 0.01 vs. Normal group. ^#^ *p* < 0.05 and ^##^ *p* < 0.01 vs. Model group. Data are presented as mean ± SEM. **Abbreviations:** GRb1, ginsenoside Rb1; GPL, gastric precancerous lesions; SEM, standard error of mean.

To further confirm the effects of GRb1 on the downstream target genes of β-catenin, such as c-myc, cyclin D1, c-jun, Wisp1 and Birc5 that are closely associated with cellular proliferation and apoptosis, RT-PCR was employed to define the transcriptional levels of these target genes. The protein levels of these target genes were determined by Western blot. It appeared that mRNA and protein levels of the genes detected were significantly higher in the GPL rats than in the normal controls. When compared with the GPL rats, mRNA levels of c-myc, cyclin D1 and Birc5 were significantly down-regulated in the GRb1-treated GPL rats (Figures 5G–K), and protein expression levels of c-myc, cyclin D1, Wisp1 and Birc5 were markedly down-regulated in the GRb1-treated GPL rats (Figures 5A–F). Thus, our findings indicated that GRb1 repressed the transcriptional activity of the target genes of β-catenin/TCF4 activation, especially c-myc, cyclin D1 and Birc5.

### GRb1 Inhibits the Hyper-Proliferation of Epithelial Cells and Accelerates Apoptosis

**FIGURE 6:**
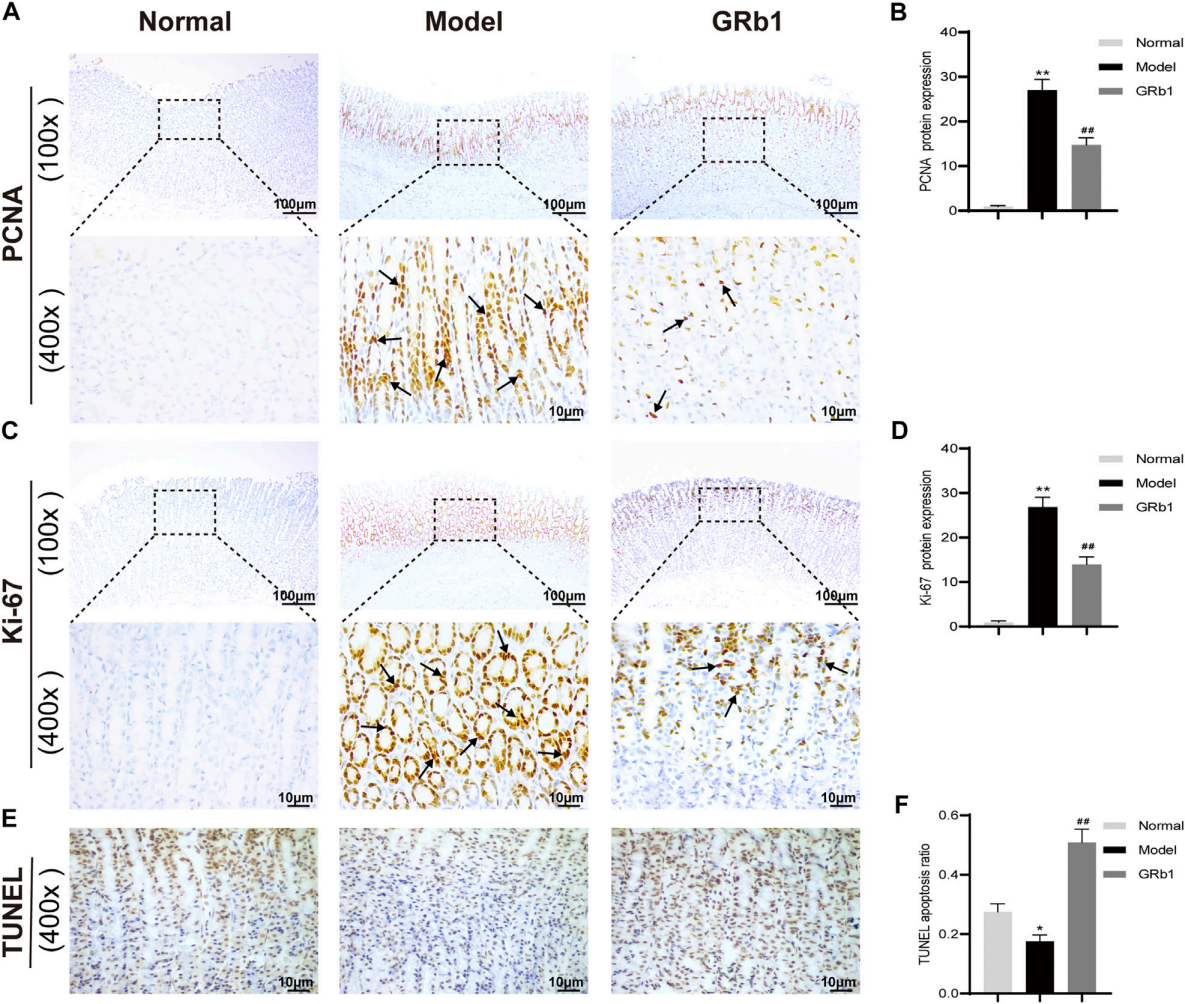
GRb1 inhibits hyper-proliferation of epithelial cells and accelerates apoptosis in GPL model rats. Representative images showing **(A)** PCNA expression and **(C)** Ki-67 expression as well as **(E)** TUNEL apoptotic cells in gastric tissue sections from each group (magnification ×100 and ×400). Semi-quantitative analysis of **(B)** PCNA protein expression levels and **(D)** Ki-67 protein expression levels as well as **(F)** TUNEL apoptosis ratio in each group (*n =* 10). *p < 0.05 and **p < 0.01 vs. Normal group. ^#^ *p* < 0.05 and ^##^ *p* < 0.01 vs. Model group. Data are presented as mean ± SEM. **Abbreviations:** GRb1, ginsenoside Rb1; GPL, gastric precancerous lesions; PCNA, proliferating cell nuclear antigen; TUNEL, TdT-mediated dUTP nick-end labeling; SEM, standard error of mean.

The proliferation and apoptosis of gastric epithelial cells was further evaluated. Firstly, the protein expression of PCNA and Ki-67, which are widely considered as the specific reporters for cell proliferation, were determined. Our IHC analysis demonstrated that the expression of PCNA and Ki-67 was significantly higher in GPL model rats than in the normal controls, suggesting that GPL rats were experiencing hyper-proliferative state of epithelial cells relative to normal rats; while GPL rats treated with GRb1 achieved a significant decrease in the expression of PCNA and Ki-67 proteins (Figures 6A–D). We then attempted to probe the apoptosis ratio of epithelial cells. From the TUNEL assay analysis, it is evident that GPL model rats displayed a lower percentage of apoptotic cells than normal rats. The apoptosis ratio was increased following GRb1 treatment (Figures 6E,F). Collectively, these findings suggested that GRb1 was able to inhibit the hyper-proliferation of epithelial cells and accelerate apoptosis in GPL rats.

## Discussion

In the present study, we found that GRb1 administration reverse intestinal metaplasia and a portion of dysplasia in the MNNG-induced GPL rats. To screen targeting DEPs and investigate the possible mechanism, we applied antibody array assay and screened out seven DEPs in GPL model group relative to normal group, in which three DEPs (β-catenin, beta-NGF and FSTL1) were significantly down-regulated after GRb1 administration. Among the three DEPs, β-catenin was the therapeutic target we were most interested in, because our earlier study showed that an herbal formula Weipixiao, of which GRb1 is the major bioactive constituent (Zeng et al., 2016), could also down-regulate β-catenin expression in GPL rats.

β-catenin is an integral structural component of cell adherens junctions and a key downstream effector of canonical Wnt pathway (Valenta et al., 2012). It has been reported that positive rate of β-catenin expression was higher in precancerous gastric tissues than and chronic non-atrophic gastritis tissues (Sun et al., 2102). Activation of β-catenin signaling by Trefoil factor 1 (TFF1) loss was proved to promote cell proliferation and gastric tumorigenesis (Soutto et al., 2015). It is worth noting that nuclear localization of β-catenin is involved in tumorigenesis. A study focusing on precancerous change in oral leukoplakia revealed that β-catenin was primarily expressed at the cell membrane in normal oral epithelium, whereas nuclear expression of β-catenin was found in 92% of oral leukoplakia with dysplasia (Ishida et al., 2007). In line with the above findings, we demonstrated that the rate of nuclear translocation of β-catenin was significantly higher in the human GPL specimens than in the healthy controls. Similar results were observed in our animal study. These lines of evidence indicate that nuclear accumulation of β-catenin plays a crucial role during malignant transition of gastric epithelium. In this study, we showed that GRb1 decreased β-catenin accumulation and localization to the nucleus in GPL rats. Similar results were reported in a recent study that GRb1 suppressed β-catenin nuclear translocation in vascular smooth muscle cells, thereby contributing to the relief of vascular calcification associated to chronic kidney disease (Zhou et al., 2019).

It has been recognized that Wnt/β-catenin/TCF-4 pathway is often aberrantly activated in gastric cancer (Zhuang et al., 2106), in which β-catenin/TCF-4 interaction occupies an important role. Upon activation of the Wnt/β-catenin signaling pathway, Wnt binds to the receptor family of curly proteins, resulting in the dissociation and accumulation of β-catenin in the cytoplasm. Free β-catenin in the nucleus binds to TCF and regulates the transcription of target genes (Pai et al., 2017). We thus interested in how GRb1 administration influenced the β-catenin/TCF-4 complex, which remain largely unexplored. We found in this research that β-catenin/TCF-4 complex was aberrantly activated in precursors of gastric cancer, and that GRb1 treatment disrupted the interaction of β-catenin with TCF-4.

Previously, increased transcription levels of c-myc, cyclin D1, c-jun, Wisp1 and Birc5 in the development and progression of gastric cancer were reported (Valenta et al., 2012; Gurbuz and Chiquet-Ehrismann, 2015; Peng et al., 2016; Liu et al., 2019). As expected, transcriptional levels of the five target genes were up-regulated in GPL model rats. GRb1 treatment down-regulated the transcription levels of c-myc, cyclin D1 and Birc5. Moreover, GRb1 treatment was found to inhibit the hyper-proliferation of epithelial cells and accelerate apoptosis in GPL rats. However, GRb1 administration showed no significant regulatory effects on c-jun and Wisp1 mRNA levels. One possible contributor is that c-jun and Wisp1 may not be the potential therapeutic targets for GPL treated with GRb1. Overall, our work demonstrated the benefits of GRb1 treatment for the management of gastric precancerous lesions, which is encouraging. However, only a single dose of GRb1 was administered, and the detailed pharmacodynamics of GRb1, such as dose-effect relationships, will be researched soon.

In summary, we showed the novel finding that GRb1 prevented the occurrence and progression of gastric precancerous lesions. The therapeutic effects might be contributed by decreasing protein expression and nuclear translocation of β-catenin and interfering with β-catenin/TCF4 interaction, and by repressing the transcriptional activity of downstream genes including c-myc, cyclin D1 and Birc5.

## Data Availability

The original contributions presented in the study are included in the article/supplementary material, further inquiries can be directed to the corresponding authors.
